# Natural Rubber (NR) Latex Films with Antimicrobial Properties for Stethoscope Diaphragm Covers

**DOI:** 10.3390/ma15103433

**Published:** 2022-05-10

**Authors:** Norfatirah Muhamad Sarih, Kevin Gwee, Simon Maher, Azura A. Rashid

**Affiliations:** 1 School of Materials and Mineral Resources Engineering, Universiti Sains Malaysia, Engineering Campus, Nibong Tebal 14300, Penang, Malaysia; fatirahsarih@usm.my (N.M.S.); kevingweegg@gmail.com (K.G.); 2 Department of Electrical Engineering and Electronics, University of Liverpool, Liverpool L69 3GJ, UK; s.maher@liverpool.ac.uk

**Keywords:** natural rubber latex, antimicrobial, mangosteen peel, zinc oxide nanoparticles, povidone-iodine

## Abstract

Systematic disinfection of the stethoscope diaphragm is required to ensure that it does not act as a vector for cross-transmission of health-related diseases. Thus, an antimicrobial latex film could be used as a cover to inhibit pathogenic bacteria from growing on its surface. The aim of this work is to determine the antimicrobial activity and mechanical properties of antimicrobial natural rubber (NR) latex films with different types of antimicrobial agents (mangosteen peel powder (MPP), zinc oxide nanoparticles (ZnO NP), and povidone-iodine (PVP-I)). The antimicrobial loading was varied from 0.5, to 1.0, and 2.0 phr to monitor the effective inhibition of Gram-negative bacteria and fungi growth. For MPP and PVP-I antimicrobial agents, a loading of 2.0 phr showed good antimicrobial efficacy with the largest zone of inhibition. Simultaneously, ZnO NP demonstrated excellent antimicrobial activity at low concentrations. The addition of antimicrobial agents shows a comparable effect on the mechanical properties of NR latex films. In comparison to control NR latex film (29.41 MPa, 48.49 N/mm), antimicrobial-filled films have significantly greater tensile and tear strengths (MPP (33.84 MPa, 65.21 N/mm), ZnO NP (31.79 MPa, 52.77 N/mm), and PVP-I (33.25 MPa, 50.75 N/mm). In conclusion, the addition of antimicrobial agents, particularly ZnO NP, can be a better choice for NR latex films because they will serve as both an activator and an antimicrobial. In a clinical context, with regard to frequently used medical equipment such as a stethoscope, such an approach offers significant promise to aid infection control.

## 1. Introduction

A stethoscope, as the universal tool of the medical profession, is frequently used for the cardiopulmonary assessment of the patient and monitors the low- and high-frequency sounds associated with the heart [1,2]. It is crucial because it provides diagnostic information and prognostic details with repeatable and non-invasive monitoring through auscultation. Therefore, the stethoscope aids in establishing good communication and allows efficient medical assessment between the medical profession and the patient [3]. However, the stethoscope diaphragm has been recognized as a potential vector for bacterial transmission because it is used in direct contact with different kinds of patients daily during diagnosis [4,5,6]. A contaminated stethoscope diaphragm can potentially transmit pathogenic organisms or microbes from one patient to another after a minimum of 3 s of contact, causing cross-contamination. This is likely to occur because the auscultation procedures typically involve several minutes of contact with the patients’ intact skin, which provides enough opportunities for pathogen transfer [7]. This poses a public health risk, especially for those who are deemed to be more vulnerable, such as immunocompromised patients [8]. Thus, the stethoscope can be considered one of the sources of healthcare-associated infections (HAI), also known as nosocomial infections.

The potential pathogenic organisms discovered at the stethoscope diaphragm surface include methicillin-sensitive and methicillin-resistant *Staphylococcus aureus*, *Escherichia coli*, vancomycin-resistant Enterococcus, *Pseudomonas aeruginosa*, and *Clotridiodes difficile* [7,8]. According to a study conducted in the Department of Pediatrics, Kasturba Medical College and Hospital, seven different genera of microorganisms were found from 43 stethoscopes used by healthcare personnel. The largest microorganism colony found on a stethoscope diaphragm was of coagulase-negative staphylococci (41.86%), followed by coagulase-positive staphylococci, *Escherichia coli*, *Klebsiella* species, *Enterococci* species, *Acinetobacter* species, and *Candida albicans* [1]. Although regular disinfection of stethoscopes is advised, this practice may not necessarily be carried out. Studies have shown that health professionals might not be committed to the routine cleaning process of the stethoscope diaphragm even though it could decrease the bacterial load. It occurs mainly when medical personnel conducts successive examinations during hospital rounds or in complicated situations [9]. Based on the investigation by Bansal and co-workers, of the 62 healthcare providers involved in the survey regarding stethoscope cleaning frequency, six individuals cleaned their stethoscopes less than once a week, six cleaned their stethoscopes every 1 to 4 weeks, two participants cleaned their stethoscopes every 5 to 8 weeks, and 15 cleaned their stethoscopes after more than eight weeks. The majority of healthcare providers who responded, 33 (53.22%), had never cleaned their stethoscopes. Most notably, none of them would clean their stethoscopes after examining each patient [5].

Muniz et al. (2012) found that 76% of 3208 healthcare providers at the Children’s Hospital Boston acknowledged that infection transmission might occur via a stethoscope. However, only 24% of providers cleaned their stethoscopes after each usage. This low prevalence of stethoscope disinfection could be a result of a lack of readily available equipment or supplies for disinfection. Other possible explanations include a lack of time, the difficulty of the task, and the absence of a clear reminder to clean the stethoscope [10]. Hence, maintaining a hygienic stethoscope diaphragm is essential to prevent cross-transmission of nosocomial infections from patient to patient.

Besides regular disinfection of the stethoscope diaphragm, the surface of the stethoscope diaphragm can be covered with an antimicrobial surface that avoids direct contact between the surface and the patient’s skin during an auscultation procedure. In that case, it can reduce the possibility of the bacteria colony transferring onto the stethoscope diaphragm. Therefore, using an antimicrobial diaphragm cover could prevent the colonisation of pathogens and microbes and hence reduce the cross-infection risk to subsequent patients [9]. Recently, during the COVID-19 pandemic and its uncertainties, it has been necessary for medical authorities to adopt adequate medical equipment and environmental disinfection rules [11,12], because a contaminated stethoscope may possibly endanger patients’ and physicians’ safety due to the potential of coronavirus to live on different surfaces for an extended length of time [13]. To overcome this, Kalra et al. suggested that stethoscope disinfection or the use of disposable barriers is necessary between every patient visit [14].

A disposable stethoscope diaphragm cover can act as a barrier to ensure good hygiene by providing physical protection from nosocomial pathogens, thus reducing the transmission of microbes [8]. In this work, a reusable stethoscope diaphragm cover with antimicrobial properties has been investigated. This research used natural rubber (NR) latex films to produce stethoscope diaphragm covers due to their outstanding flexibility and mechanical strength. NR latex is a renewable natural resource extracted from the rubber tree (*Hevea brasiliensis*) [15]. The main advantage of using NR latex as a diaphragm cover is that it can be stretched easily over the diaphragm and readily manipulated during installation. Besides, it can be made into a thin and smooth film that provides good barrier properties against blood-borne pathogens and viruses [16,17]. The thickness of the film can be easily controlled by adjusting the dwell time during the latex dipping process [18]. Thin-film formation is vital to maintain adequate sound transmission during the auscultation process. Furthermore, NR latex can be compounded with antimicrobial agents to suppress the growth of nosocomial pathogens.

An antimicrobial agent is a natural or synthetic substance that can kill or inhibit the growth of microorganisms by disrupting the essential microbial metabolic pathway or changing the cell membrane or cell wall structure [19]. There are a wide variety of antimicrobial agents that have proven their antimicrobial activities in NR latex, such as chitin [20], mangosteen peel powder [21,22], zinc oxide nanoparticles [23,24], silver nanoparticles [25], vegetable oil emulsion [26], and povidone-iodine [27]. Each antimicrobial agent has its mechanism for suppressing the growth of pathogenic microorganisms. However, a comparison of their antimicrobial efficiency concerning certain species of microorganisms has not been investigated yet to the best of our knowledge. Further, the addition of antimicrobial agents should not diminish the mechanical properties of the NR latex films. An antimicrobial NR latex film needs to exhibit good antimicrobial activity and structural integrity to withstand the stretching process during installation and removal on the stethoscope diaphragm. Therefore, it is essential to obtain a high antimicrobial activity of NR latex films while maintaining sufficient mechanical strength [28,29].

This research involved the preparation of the antimicrobial NR latex films for a stethoscope diaphragm covered with different antimicrobial agents (mangosteen peel powder (MPP), zinc oxide nanoparticles (ZnO NPs), and povidone-iodine (PVP-I)). The main goal was to investigate the antimicrobial activity and mechanical properties of NR latex films with the antimicrobial agents mentioned. Furthermore, the impact of post-processing on antimicrobial activity and mechanical properties of the antimicrobial NR latex films was investigated after being subjected to leaching and accelerated ageing processes to ensure the stability of the films based on the antimicrobial activity and mechanical properties exhibited.

## 2. Materials and Methods

### 2.1. Materials

The natural rubber (NR) latex used was a high-ammonia NR latex (HA-NR latex) that was purchased from Zarm Scientific (M) Pte. Ltd. (Penang, Malaysia). Zinc Oxide nano-powder was used with less than 100 nm particle size and 81.39 g/mol molecular weight (purchased from the Sigma-Aldrich Corporation, St. Louis, MO, USA). Polyvinylpyrrol-idone iodine (PVP-I) used was 10% *w*/*v* of povidone-iodine (the equivalent of 1% *w*/*v* available iodine) purchased from Dynapharm (M) Sdn Bhd (Penang, Malaysia) and used as received. Other chemicals, including sulphur, zinc diethyl dithiocarbamate (ZDEC), zinc oxide (ZnO) dispersions, potassium hydroxide (KOH), and antioxidants were purchased from Farben Technique (M) Sdn Bhd., Penang, Malaysia and used as received. Potassium laureate with 20% total solid content (TSC) was prepared from a mixture of lauric acid and KOH.

### 2.2. Preparation of Antimicrobial NR Latex Films

A total of 450 mL NR latex compound was prepared. As shown in Table 1, the compounding ingredients were weighed and prepared in separate beakers according to the formulation. For the NR latex mixtures M, Z, and P, each loading of the antimicrobial agent was repeated for 1.0 and 2.0 phr. The weighed NR latex was subjected to mechanical stirring at 270 rpm. During stirring, the weighed compounding ingredients were added into the NR latex with a consistent compounding sequence: potassium laurate → KOH → ZnO → antioxidant → antimicrobial agent → ZDEC → sulphur. The NR latex mixture was diluted with distilled water until the final TSC was around 40 ± 1%. The NR latex mixture was stirred for two hours to achieve homogeneity of the compounding materials. After thorough mixing, the NR latex mixture was pre-vulcanised in a water bath at 70 ± 1 °C for 1 h with continuous stirring. The NR latex mixture was sealed with aluminum foil to prevent contamination during the compounding procedure. Prior to vulcanisation, the NR latex compound was tested for chloroform number. At 5-min intervals, 5 cm^3^ of NR latex was taken and mixed with an equivalent volume of chloroform using a glass rod until coagulation was complete. Based on physical properties, the coagulum was evaluated and rated. At chloroform number 2, the pre-vulcanisation process was halted. The NR latex compound was sealed and allowed to mature for 24 h at room temperature.

A filter was used to remove contaminants and residues from the NR latex compound before sieving it into a latex dipping tank. The porcelain dipping plates were adequately cleaned with soap and water. The dipping plates were dried in an air oven at 70 °C for about 10 min to make sure they were dry. The coagulant dipping tank was filled with a 10% calcium nitrate solution coagulant. The dried plates were immersed in the coagulant for 10 s and then dried in an oven set at 70 °C for 10 min. After 10 s of dwell time, the dry plates were removed from the oven and dipped into the NR latex compound. The dipping plates were carefully withdrawn to ensure a consistent latex deposit. The NR latex films were cured for 30 min at 100 °C in an air oven. Calcium carbonate powder was used to help remove the NR latex films from the porcelain plate to avoid sticking to the porcelain. After removing the NR latex films from the oven, they were allowed to cool to room temperature for 24 h before stripping.

### 2.3. Post-Processing of NR Latex Film

Wet-gel leaching, dry-gel leaching, and a combination of wet and dry gel leaching were all used in the leaching process. For wet-gel leaching, after latex dipping, the dipping plates were held at room temperature for 10 min before being wet-gel leached for 1 min in distilled water at 70 °C. Subsequent wet-gel leaching, vulcanisation, and stripping of the NR latex films were performed. Dry-gel leaching was performed by soaking the NR latex films in distilled water for 24 h at room temperature after they were stripped from the plates. For the combination of wet and dry-gel leaching, the NR latex films were pre-leached in distilled water at 70 °C for 1 min before vulcanisation. After stripping, the NR latex films were re-leached for 24 h at room temperature in distilled water. The accelerated ageing was carried out according to ASTM D573. The NR latex films were aged at a temperature of 100 °C in an air oven for 3 consecutive days.

### 2.4. Preparation of Bacteria

A soil dilution method with 1:10 serial dilution was used to isolate soil bacteria and reduce the number of soil bacterium species. Soil is a media rich with bacteria, providing a simple, low-cost and convenient means to collect a wide range of bacteria including the likes of *Escherichia coli* [30,31,32,33,34], *Pseudomonas aeruginosa* [35,36,37], *enterococci* [30,31,32,38] and *Clostridioides difficile* [39,40,41,42,43] which are undesirable within clinical settings. Initially, the soil sample was sieved to eliminate any particles, roots, or stones. After weighing 1 g of soil, it was added to a conical tube containing 9 mL of distilled water. After that, the conical tube was vigorously shaken to mix the solution. The three sterile test tubes were labeled 10^−2^, 10^−3^, and 10^−4^, respectively. Using a transferpette, 9 mL of distilled water was added to each test tube. Then, 1 mL of the soil solution was transferred from the conical tube to the test tube labeled with 10^−2^ and gently swirled until the solution was well mixed. By transferring 1 mL of the solution to the subsequent test tube, this step was repeated for the test tubes labeled with 10^−3^ and 10^−4^. The bacterial solution in the 10^−4^ test tube was then utilised for agar disc diffusion.

### 2.5. Characterisation of Antimicrobial Latex Film

#### 2.5.1. Antimicrobial Test

The antimicrobial activity testing of the latex films was conducted using the agar diffusion method by determining the zone of inhibition. First, 4.95 g of MacConkey (MCA) agar powder was weighed and transferred to a sterile conical flask. The flask was then filled with 75 mL deionised water and stirred using a magnetic stirrer. Then, 25 mL deionised water was added to the conical flask after two minutes of stirring. It was then allowed to cool to between 40 °C and 50 °C before being poured into individual Petri dishes. The latex films were cut into a diameter of 20 mm and dipped in the agar before its solidification. A glass rod was used to streak 100 μL of the bacterial solutions onto the agar plate. After a week at room temperature, the plates were observed for the zone of inhibition (mm).

#### 2.5.2. Tensile Test

The tensile test was carried out according to the ASTM D416 standard. The NR latex films were cut into a dumbbell shape according to the direction of dipping by using a Wallace die cutter. Five dumbbell test pieces were prepared for each compound. The thickness of each test piece was measured at three different points using a thickness gauge, and the average thickness was taken. The tensile test was carried out using the Instron machine with a 500 mm/min crosshead speed until the test pieces failed. Then, the tensile properties such as tensile strength, elongation at break, and tensile modulus (M100, M300, and M500) were obtained and their average readings were recorded.

#### 2.5.3. Tear Test

The tear test was conducted according to the ASTM D624 standard. The NR latex films were cut into angle test specimens based on the dipping direction. The thickness of each test piece was measured using a thickness gauge and the average thickness was taken. The tear test was carried out using an Instron machine with a crosshead speed of 500 ± 50 mm/min until the test pieces’ failure. The tear strength was obtained, and the average readings were recorded.

#### 2.5.4. Swelling Test

The swelling test was conducted according to the ASTM D471 standard. The NR latex films were cut into 2 cm × 2 cm dimension test pieces. The initial weight of each test piece was weighed using an electronic weighing balance. The test pieces were then immersed in a glass bottle containing toluene for 24 h at room temperature. The glass bottle was closed tightly to prevent the evaporation of toluene. The test pieces were dried in between tissue papers and weighed again after 24 h. The swelling percentage, $X_{s}$, was calculated using Equation (1).

(1) $$X_{s}=\frac{W_{i}-W_{s}}{W_{i}}\times 100\%$$

where

W_i_ = initial weight of test piece;
W_s_ = swollen weight of test piece.

#### 2.5.5. Crosslink Density

The NR latex films with the swollen networks were dried in a 60 °C air oven until a constant weight was achieved. The weight of the unswollen network at equilibrium was recorded. Equation (2) was used to determine the volume fraction of latex films in their swollen state, *V_r_*.

(2) Vr=Wbρ1Wbρ1+Wa−Wbρ2×100%

where

*W_a_* = weight of swollen latex sample after toluene immersion;
*W_b_* = weight of unswollen latex sample after drying;
ρ1 = density of the latex samples;
ρ2 = density of toluene (0.867 g/cm^3^) [44].

The crosslink density, χ, of the NR latex films was then determined using Flory-Rehner Equation (3).

(3) χ=−ln1−Vr+Vr+xVr2VsVr1/3−Vr2

where

*χ* = interaction parameter for the rubber−toluene system (0.39);
*V_r_* = The volume fraction of the latex films in swollen state;
*Vs* = Molecular volume of toluene (106.2 cm^3^/mol).

## 3. Results

### 3.1. Effect of Antimicrobial Agent Loading on Antimicrobial Activity of NR Latex Films

**Figure 1:**
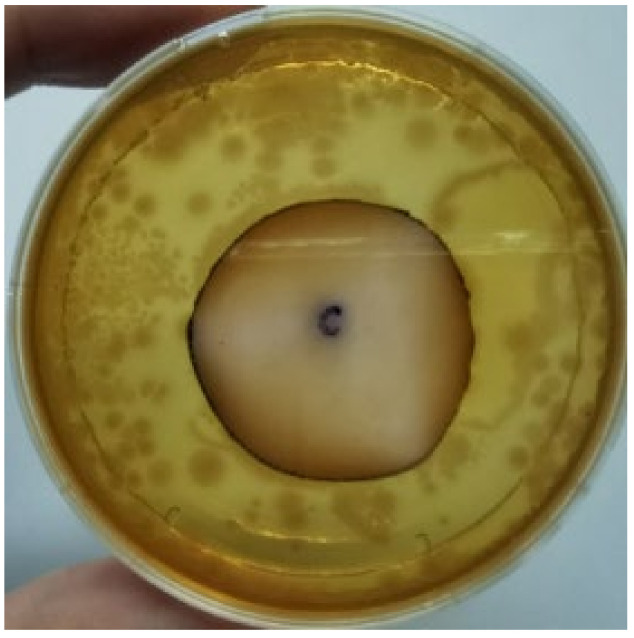
Agar disc diffusion assay of unfilled NR latex film.

The agar disc diffusion method was used to screen the unleached NR latex films for antimicrobial activity against Gram-negative bacteria. Bacterial growth on MacConkey agar is displayed in Figure 1, with the control sample immersed in the plate’s centre. Numerous bacteria (transparent colonies) were seen on the agar surface.

The agar disc diffusion of NR latex films soaked with MPP, ZnO NP, and PVP-I at various loadings is shown in Table 2. The number of bacteria colonies on nutritional agar reduced as the antimicrobial agent loading increased. It was shown that the antimicrobial ingredient may diffuse from the NR latex films into the agar and suppress bacterial growth [45]. The bioactive chemicals in mangosteen peel may disrupt the bacterial membrane by increasing the permeability of the cell membrane, resulting in the loss of cytoplasm contents [46]. The PVP-I is likely to emit free iodine, which could oxidise the essential pathogen structure and membrane components, resulting in the creation of pores and eventually cytosol leakage [47]. ZnO nanoparticles had greater antibacterial activity than normal ZnO, owing to their smaller particle size and faster ZnO dissolution to Zn^2+^ [48].

### 3.2. Effect of Antimicrobial Agent Loading on Mechanical Properties of NR Latex Films

Table 3 shows the tensile and tear strengths of unfilled and filled NR latex films with different antimicrobial agent loadings. Both MPP- and ZnO NP-filled NR latex films slightly increased tensile strength with 0.5 phr antimicrobial agent. In comparison to the control sample, the PVP-I-filled NR latex films demonstrated equivalent strength. The tensile strength of the NR latex films decreased as the MPP loading increased to 2.0 phr. Increased MPP loading of latex resulted in decreased intermolecular cohesion, which affected the network’s dilational deformation during latex film production, causing a loss of mechanical properties. On the other hand, the tensile strength of NR latex films containing ZnO NP remained remarkably stable throughout a range of loadings. It was shown that a minimal concentration of nano-sized ZnO was sufficient to maximise crosslinking formation efficiency due to their high specific surface area and reactivity. By substituting nano-sized ZnO for conventional ZnO, the mechanical strength of latex films might be increased [48]. However, increasing the loading further significantly improves the NR latex films’ tensile strength. In the case of PVP-I-filled NR latex films, raising the PVP-I concentration may increase the tensile strength of the NR latex films. Interchain crosslinking may be facilitated by various radical species, most notably allyl radicals produced during the iodination reaction [49].

The tear strength of NR latex films using ZnO NP was slightly higher than that of the control sample containing conventional ZnO. It could be because ZnO NP was distributed more uniformly in the rubber matrix than ordinary ZnO. Additionally, ZnO NP may exhibit superior interfacial characteristics and rubber-filler interaction compared to standard ZnO [24]. The inclusion of MPP and PVP-I may further improve the tear strength of NR latex films, as MPP-filled latex films demonstrated greater tear strength. It could be because the filler deviates from the cracks, resulting in non-linear crack growth. This crack deviation was considered a characteristic of filled NR latex films that added strength [46].

### 3.3. Swelling Index and Crosslink Density

**Figure 2:**
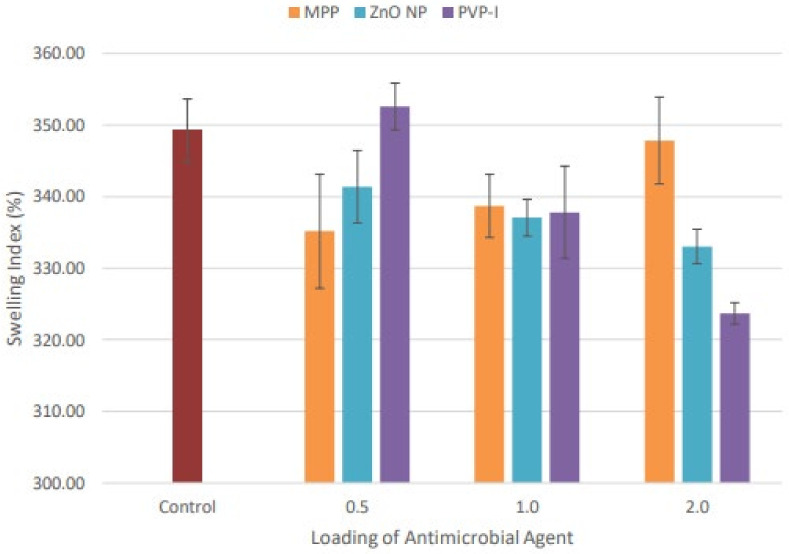
Swelling index percentage of unfilled and filled NR latex films at different antimicrobial loading. The error bars relate to the standard deviation calculated from 3 repeats.

**Figure 3:**
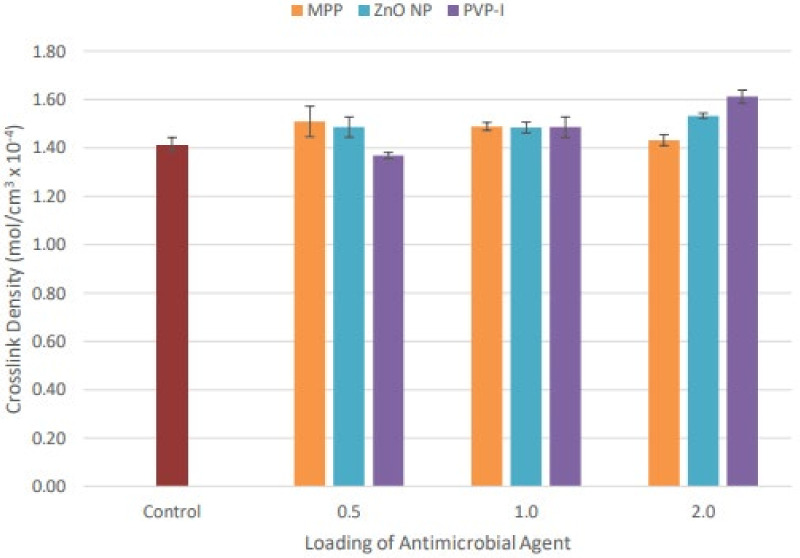
The crosslink density of unfilled and filled NR latex films at different antimicrobial loading. The error bars relate to the standard deviation calculated from 3 repeats.

The swelling index assessed the equilibrium swelling ability of NR latex films after immersion in the toluene for 24 h at room temperature. It was closely associated with the formation of crosslink density in the NR latex films. The NR latex films with a higher crosslink density were expected to have a higher resistance against swelling. Figure 2 and Figure 3 illustrated the swelling index and crosslink density of the NR latex films with varied loading of antimicrobial agents, respectively. MPP-loaded NR latex films demonstrated the lowest swelling index at 0.5 phr. The swelling index gradually increased as the loading of MPP increased, because there were fewer limits for the solvent to pass through the matrix at higher loading. The swelling index of 2.0 phr MPP-filled NR latex films was comparable with the control sample, while ZnO NP-filled NR latex films displayed a decreasing trend in the swelling index when the loading increased. This may have been due to the increased activity of ZnO NP in the crosslinking process. A high specific surface area enables a more substantial contact between the crosslinking agent and polymer chains [50]. Thus, the crosslink density increased correspondingly with the increase of ZnO NP loading, leading to a reduced swelling index. Moreover, PVP-I-filled NR latex films similarly demonstrated a decreasing tendency in the swelling index from 0.5 phr to 2.0 phr. Higher loading of PVP-I offered higher resistance to swelling in toluene. As the concentration of iodine increased, the crosslinking reaction of the iodinated chains increased, resulting in a reduced swelling index [51].

### 3.4. Effect of Leaching and Accelerated Ageing on Antimicrobial Activity of NR Latex Films

As shown in Table 4, different leaching conditions affected the antimicrobial properties of the NR latex films. After leaching, most antimicrobial NR latex films showed a slight reduction in antimicrobial efficacy in areas where fungal colonies can be visible. MPP-filled NR latex films reduced antibacterial activity more than ZnO NP- and PVP-I-filled NR latex films. Additionally, transparent bacteria colonies on MPP-filled NR latex films were observed surrounding the films, indicating a good inhibition action after wet and dry leaching.

**Figure 4:**
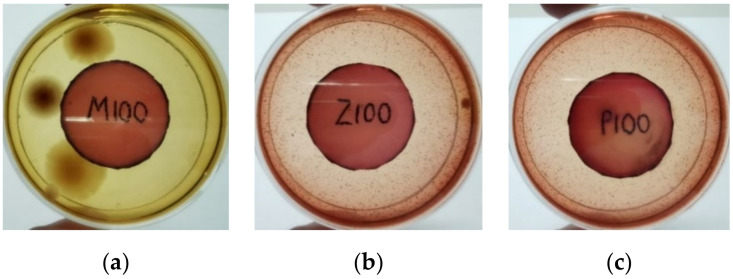
Agar disc diffusion assay of filled NR latex films after 100 °C ageing for three days. (**a**) M100 = MPP after 100 °C ageing; (**b**) Z100 = Zn NP after 100 °C ageing; (**c**) P100 = PVP-I after 100 °C ageing.

Figure 4 exhibits the antibacterial activity of the NR latex films after 3 consecutive days of accelerated ageing at 100 °C. The MPP-filled NR latex film had the most significant loss in antimicrobial activity, which contained more fungal colonies on the nutritional agar surface than the other filled NR latex films. When the mangosteen pericarp extract was heated to a high temperature for an extended period, the xanthones concentration tended to degrade enzymatically or thermally [52]. This loss of xanthones may decrease antibacterial action, resulting in increased fungal colonies after 3 days at 100 °C. After accelerated ageing, the antibacterial activity of NR latex films with ZnO nanoparticles and PVPI remained comparable to before ageing.

### 3.5. Effect of Leaching and Accelerated Ageing on Mechanical Properties of NR Latex Films

The tensile and tear strengths of the unfilled and filled NR latex films at different leaching conditions are given in Table 5. Typically, leaching might increase the tensile strength of NR latex due to elimination of water-soluble non-rubber ingredients and increasing the effective coalescence of rubber particles [49]. After leaching, the filled NR latex film demonstrated better tensile strength than the unfilled NR latex film. The tear strength of the filled NR latex films was also enhanced after the leaching procedure. A combination of wet and dry gel leaching demonstrated the optimal mechanical properties in tensile and tear strength.

**Figure 5:**
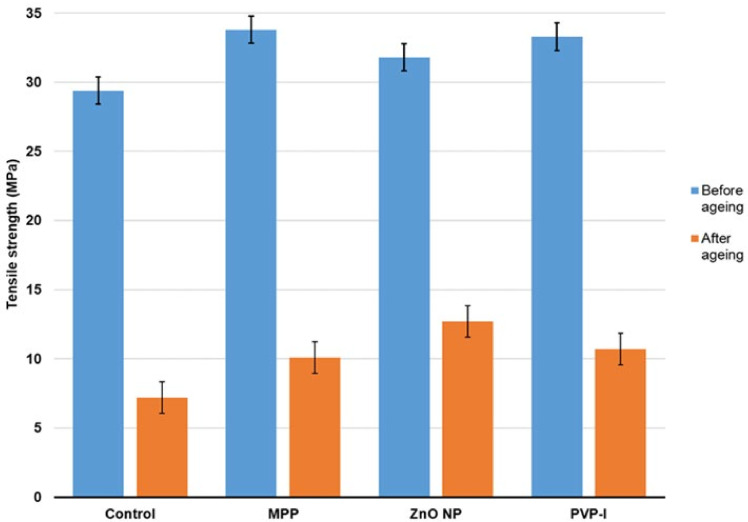
Mechanical properties of antimicrobial NR latex films before and after accelerated ageing at 100 °C. The error bars relate to the standard deviation calculated from 3 repeats.

The tensile strength of unfilled and filled NR latex films before and after ageing at 100 °C for 3 consecutive days is exhibited in Figure 5. After ageing, the filled NR latex films showed higher retention than unfilled NR latex films. Furthermore, the ZnO NP-filled NR latex films demonstrated maximum retention properties, followed by PVP-I- and MPP-filled NR latex films. This demonstrated that the inclusion of antimicrobial compounds could result in more significant retention of NR latex films’ mechanical properties when subjected to extremely high temperatures.

## 4. Conclusions

The antimicrobial agents MPP, ZnO NP, and PVP-I were proven to provide desired antimicrobial activity to the NR latex films against Gram-negative bacteria at the optimum loading of 2.0 phr. The mechanical properties of the filled NR latex films were still on par with the unfilled NR latex films. NR latex films with antimicrobial were found to have the comparable tensile strength to the control samples. Although the antimicrobial NR latex films had a higher tear strength than the control sample, this was not the case for all samples. The tensile and tear strength was not significantly affected by the addition of antimicrobial agents in concentrations ranging from 0.5 to 2.0 phr. As a result, adding antimicrobial agents to NR latex films will provide good antibacterial activity and structural stability for use as a stethoscope diaphragm cover. ZnO NP- and PVP-I-filled NR latex films reduced antibacterial activity less than MPP-filled NR latex films. After post-processing, the mechanical properties of NR latex films containing antimicrobial agents were comparable to those of the control sample. NR latex films treated with antimicrobials had mechanical properties that remained comparable with the control sample after post-processing. Antimicrobial agents, particularly ZnO NP, could be a better option for NR latex films because they can serve as activators and antibacterial agents. This proof-of-concept study has demonstrated the potential benefit of an antibacterial stethoscope-compliant cover. In future work, it is desired that the optimal ZnO NP NR latex film can be trialed in a representative clinical setting.

## Figures and Tables

**Table 1:** The formulation for pre-vulcanized NR latex compounds with the addition of 0.5 phr of antimicrobial agents.

Ingredients	Composition (Dry Weight phr)			
	C	M	Z	P
60.80% NR latex	100	100	100	100
10.00% KOH	0.8	0.8	0.8	0.8
20.00% potassium laurate	0.4	0.4	0.4	0.4
50.80% ZnO (in micro-size)	1	1	-	1
53.80% ZDEC	1	1	1	1
54.00% sulphur	2	2	2	2
56.55% antioxidant	1	1	1	1
50.00% mangosteen peel powder *	-	0.5	-	-
10.00% povidone-iodine *	-	-	-	0.5
5.80% ZnO (in nano-size) *	-	-	0.5	-
Total	106.2	106.7	105.7	106.7

* The loading was repeated for 1.0 and 2.0 phr. C = control, M = MPP, Z = ZnO NP, P = PVP-I.

**Table 2:** Agar disc diffusion assay of antimicrobial agent-filled NR latex with the loading of 0.5 phr, 1.0 phr, and 2.0 phr.

Antimicrobial Agent	0.5 phr	1.0 phr	2.0 phr
MPP	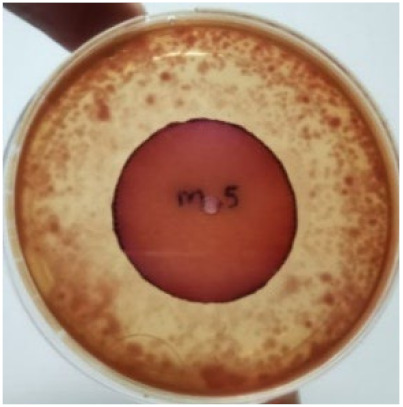	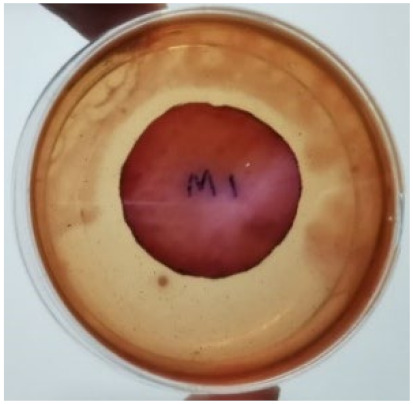	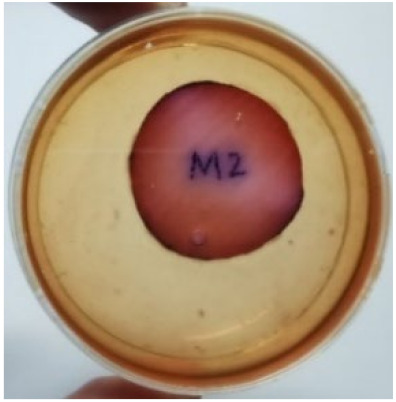
ZnO NP	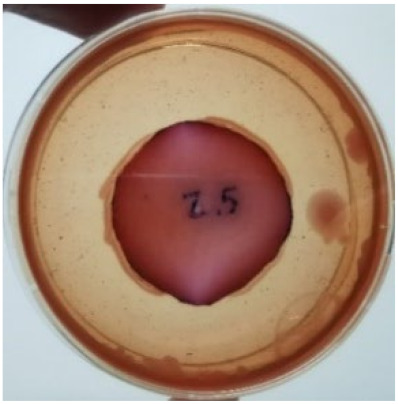	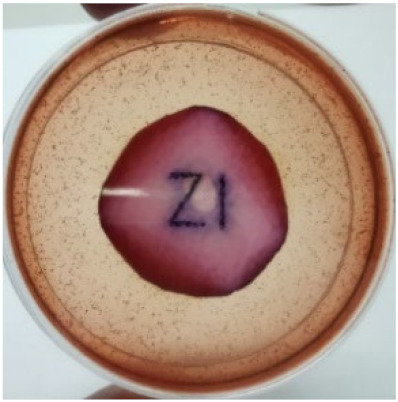	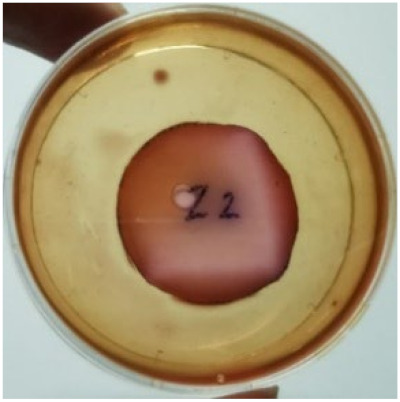
PVP-I	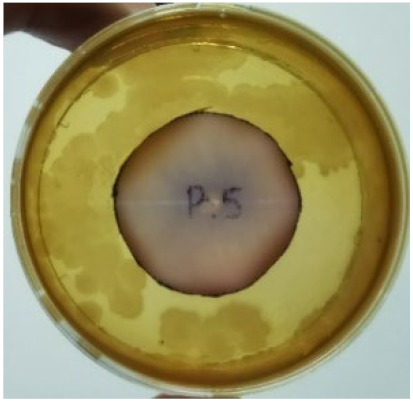	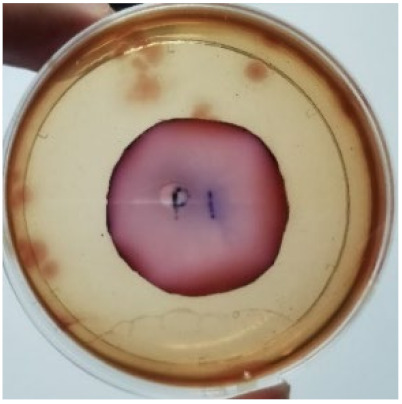	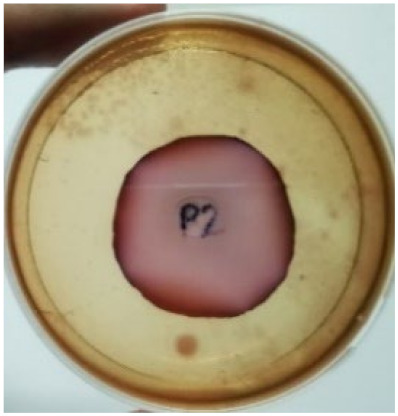

**Table 3:** Mechanical properties of antimicrobial NR latex films.

Antimicrobial Agent Loading	Control		MPP-Filled		ZnO NP-Filled		PVP-I-Filled	
	Tensile (MPa)	Tear (N/mm)	Tensile (MPa)	Tear (N/mm)	Tensile (MPa)	Tear (N/mm)	Tensile (MPa)	Tear (N/mm)
0.0	27.58	39.74	-	-	-	-	-	-
0.5	-	-	29.64	47.24	31.18	43.94	27.84	46.68
1.0	-	-	27.54	47.19	31.24	43.22	30.38	46.46
2.0	-	-	25.53	47.51	30.85	43.34	31.46	46.33

**Table 4:** Agar disc diffusion assay of antimicrobial agent-filled NR latex films after wet-gel leaching, dry-gel leaching, and wet and dry-gel leaching.

Antimicrobial Agent	Wet-Gel	Dry-Gel	Wet and Dry Gel
MPP	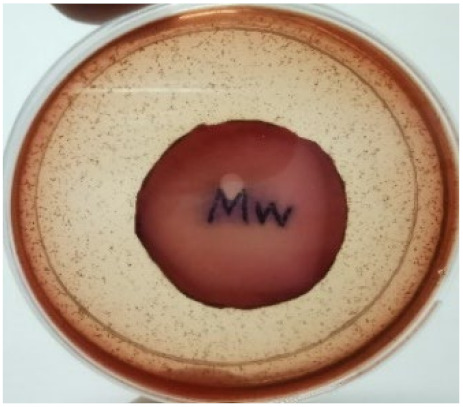	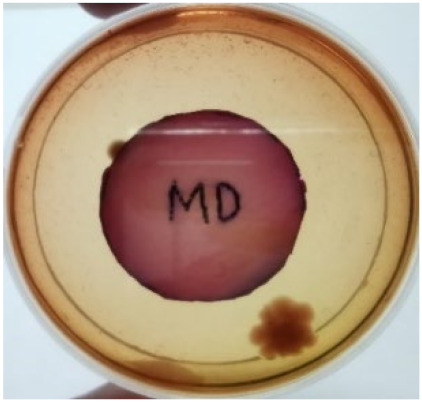	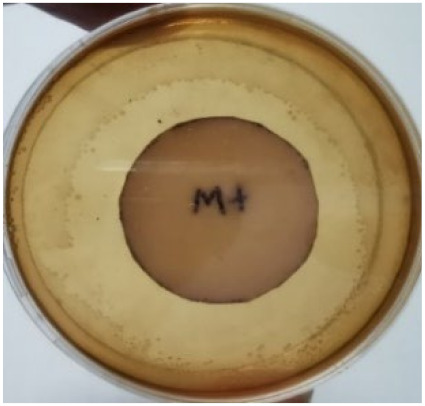
ZnO NP	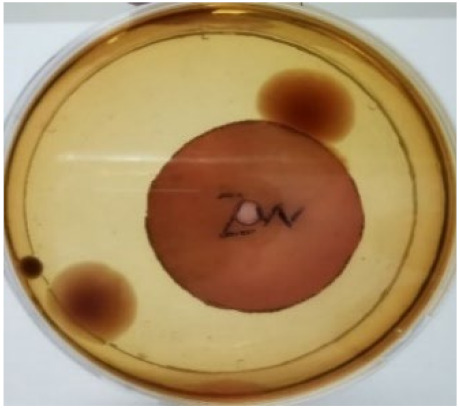	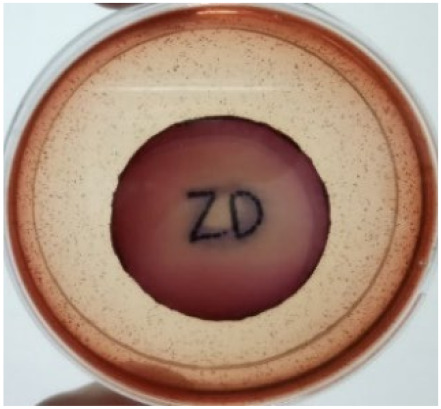	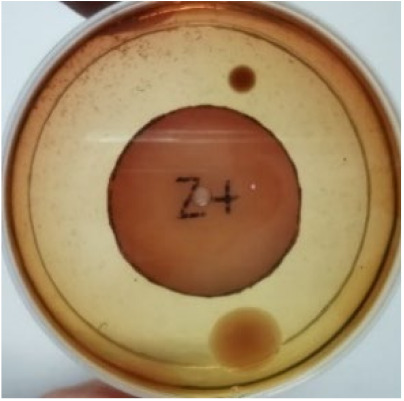
PVP-I	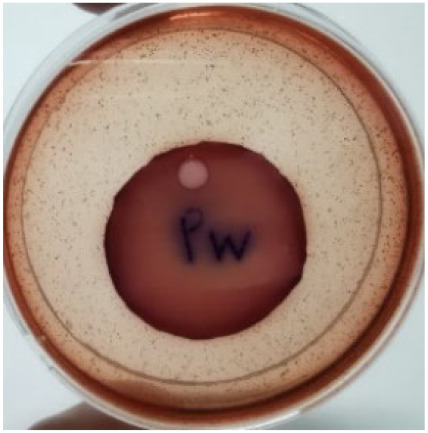	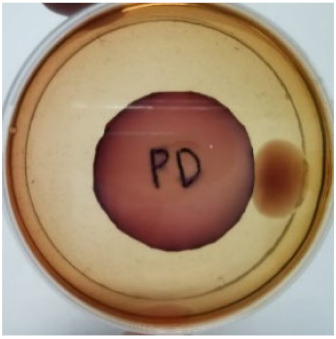	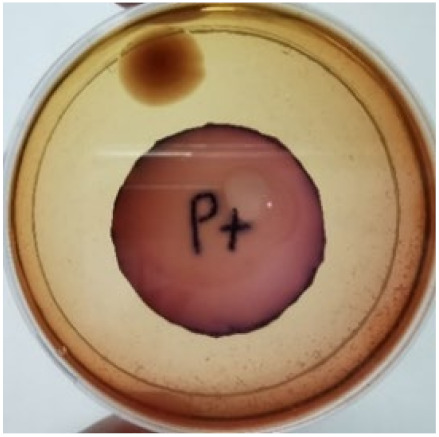

**Table 5:** Mechanical properties of antimicrobial NR latex films after leaching.

Leaching Conditions	Control		MPP-Filled		ZnO NP-Filled		PVP-I-Filled	
	Tensile (MPa)	Tear (N/mm)	Tensile (MPa)	Tear (N/mm)	Tensile (MPa)	Tear (N/mm)	Tensile (MPa)	Tear (N/mm)
Unleached	27.58	43.06	25.53	47.51	30.85	43.34	31.46	46.33
Wet	31.17	44.06	32.60	50.10	31.95	47.13	32.84	47.53
Dry	29.72	48.03	34.38	54.48	31.25	47.75	35.79	47.88
Wet + Dry	29.41	48.49	33.84	65.21	31.79	52.77	33.25	50.75

## Data Availability

All the actual data are presented in the manuscript.
